# Verbal narrative ability and episodic autobiographical memory in adolescents and young adults with 22q11.2 deletion syndrome

**DOI:** 10.1186/s11689-025-09606-8

**Published:** 2025-03-29

**Authors:** Claire Mayor, Julie Husmann, Selma Benaghmouch, Stephan Eliez, Clémence Feller, Maude Schneider

**Affiliations:** 1 Child Neuropsychology Unit, Faculty of Psychology and Educational Sciences, University of Geneva, 40, Boulevard du Pont-d’Arve, Geneva, 1205 Switzerland; 2 Clinical Psychology Unit for Intellectual and Developmental Disabilities, Faculty of Psychology and Educational Sciences, University of Geneva, 40, Boulevard du Pont-d’Arve, Geneva, 1205 Switzerland; 3 Developmental Imaging and Psychopathology Lab Research Unit, Faculty of Medicine, University of Geneva, Geneva, Switzerland; 4 Department of Genetic Medicine and Development, Faculty of Medicine, University of Geneva, Geneva, Switzerland

## Abstract

**Background:**

Poor episodic autobiographical future thinking has recently been reported in 22q11.2 carriers. However, whether these impairments are due to poor language skills or indicate a true episodic autobiographical memory deficit remains unclear. Language impairments are the hallmark of the neuropsychological profile of young children with 22q11DS, but language outcomes in adolescence and young adulthood, especially high-level linguistic skills such as narrative, remain largely unexplored. The aims of this study are first to precisely characterize the narrative abilities of a group of adolescents and young adults with a 22q11DS and normal verbal intellectual functioning, in comparison to a control group. Second, to assess their (past) autobiographical episodic memory and their future episodic thinking abilities. Third, to examine the relationship between linguistic and autobiographical memory skills.

**Methods:**

Fifteen adolescents and young adults with 22q11DS were compared with 15 age- and sex-matched controls. Narrative ability was assessed with a storytelling task and included microstructural, macrostructural, and pragmatic linguistic measures. Episodic autobiographical memory was assessed using a paradigm involving recall of past personal memories and future thinking conditions.

**Results:**

Adolescents and young adults with 22q11DS still struggled with high-level language skills such as storytelling tasks, and all linguistic levels were impaired, i.e., the microstructural, macrostructural, and pragmatic components of narrative. Second, 22q11DS carriers showed poor episodic autobiographical recall of their personal memories and reduced access to sensory details (visual, auditory…) compared to controls. Their poor autobiographical episodic memory skills were independent of language impairment, and there were no effects of age or intellectual level on their autobiographical (past) memories recollection. On the other hand, age and verbal intellectual functioning significantly contributed to their ability to produce episodic narratives in the future thinking condition, suggesting that the future thinking task relies on more complex and intricate factors than pure episodic memory ability.

**Conclusions:**

Verbal narrative impairments did not account for poor recall of personal memories, suggesting dysfunctional episodic memory networks between hippocampi and posterior cortical areas in 22q11DS, where neuroanatomical and neurofunctional alterations have indeed been reported.

## Background

Velo-cardio-facial syndrome or 22q11 deletion syndrome (22q11DS) is a genetic syndrome that combines physical affections (primarily heart defects and submucosal cleft palate), cerebral peculiarities, neuropsychiatric and neurocognitive disorders [1, 2] with particular developmental trajectories [3].

From a neuropsychological standpoint, 22q11DS carriers typically present with borderline intellectual functioning (mean normal TIQ distribution around 70), with a superiority of the verbal quotient over the nonverbal quotient, attentional-executive deficits [4, 5], and visuospatial processing disorders [6] suggestive of a pattern of nonverbal learning disabilities [7, 8]. Furthermore, a hallmark of the syndrome is delayed language development from an early age, as more than 95% of children with 22q11.2DS have delayed early language milestones, with persistent deficits throughout childhood [9]. Thus, a late onset of first words and sentences with persistent language delays in school-aged children is reported [9–14]. Language onset delays appear to be more severe in young children with intellectual disability than in those with preserved intellectual functioning [15]. However, the issue of language skills in 22q11DS carriers with preserved intellectual functioning for older school-age children, aged 10–11 years, remains controversial. Indeed, Roizen et al. [10] demonstrated persisting language impairments in children with preserved intellectual functioning, while Vicari et al. [16] found no differences in lexical and morphosyntactic language skills when comparing control children and 22q11DS carriers with preserved intellectual functioning. On the other hand, while language production seems to be more affected than comprehension in young children, this relative difference in the strength of abilities then seems to diminish over time [17] or even reverse itself, with expressive skills becoming superior to comprehension skills [13, 18].

In these studies, language skills are based on standardized lexical and morphosyntactic tests. However, these tests do not capture higher-level, more complex language skills, such as narrative skills. Therefore, they may not be sensitive enough to detect expressive narrative difficulties, especially in older participants. Telling a story requires a good mastery of all linguistic levels. Narrative skills indeed involve managing the macro-structure of the narrative (narrative stages including an introduction, a trigger for complication, the search for solutions, twists and turns, the resolution of the problem and the conclusion), but also the micro-structure, i.e. the appropriate use of morphosyntax and grammar, as well as the choice of lexicon. Finally, narrative requires pragmatic skills, including inference and theory of mind. Narration provides much more information than most standardized language tests, making it an ideal task for assessing complex linguistic processes [19]. Only two studies have examined the narrative abilities of children with 22q11DS, showing deficits in production in a group of 5 to 8 years olds [14] and in comprehension for the 8 ½ years olds [18]. In the latter study, comprehension of the narrative’s macrostructure was not explained by mental level, and participants with 22q11DS showed similar scores to participants with developmental language disorders (DLD). To date, no data are available on the narrative abilities of adolescents and young adults with 22q11DS.

On the other hand, poor episodic autobiographical future thinking has recently been reported in 22q11.2 carriers [20]. However, whether these impairments are due to poor language skills or indicate a true episodic autobiographical memory deficit remains unclear. Language, and more specifically narrative skills are indeed solicited when recalling autobiographical memories [21]. In 22q11 DS carriers, superiority of verbal over visuospatial episodic memory skills has been reported, with verbal memory situated in the low average range [22, 23]. Recently, however, accelerated forgetting rate [24] and reduced verbal learning [25] have been reported and these memory results correlated with the level of psychotic features. However, to date, long-term memory has mostly been assessed using word list learning tasks, and autobiographical episodic memory in participants with a 22q11DS remains largely unexplored. To our best knowledge, only one study examined episodic autobiographical memory with a future episodic projection task in 22q11DS carriers (adults), and showed that the narratives were shorter and contained fewer details compared to controls, resulting in autobiographical narratives that lacked overall “episodicity” [20].

The aims of this study are, first to precisely characterize the narrative abilities of a group of adolescents and young adults with a 22q11DS and a normal verbal intellectual quotient/functioning (VCI > 70) in comparison to a control group. Second, to assess their autobiographical episodic memory and the future episodic projection abilities. Third, to examine the relationship between narrative and autobiographical memory skills in order to better understand whether the future episodic memory impairments reported in a previous study are due to poor language skills or to true autobiographical memory deficits.

Although literature reports the normalization of basic lexical and morphosyntactic skills during late childhood in 22q11 DS carriers, we expect persistent complex linguistic impairments, i.e. poor macrostructure and pragmatic mastery in our group of adolescents and young adults. On the other hand, we expect to replicate the previous results showing impaired autobiographical memory [20] but according to the importance of narrative skills in autobiographical memory, we expect to find a role of linguistic mastery in autobiographical memory results.

## Methods

### Participants

Thirty participants between 12 and 30 years of age were included (mean age = 21.78, SD = 5.6). Fifteen participants were 22q11DS carriers and were recruited from the Swiss 22q112DS cohort. All participants in the 22q11DS group had a confirmed genetic diagnosis of 22q11.2 microdeletion. Fifteen control participants were individually matched for age and gender and recruited from university advertisements or through acquaintances.

Inclusion criteria for all participants were [1] adequate French language skills, and [2] age between 12 and 30 years. Inclusion criteria for 22q11DS participants was a verbal intellectual quotient (as measured with the verbal comprehension index, VCI) on the Wechsler Intelligence Scale within the normal range.

Exclusion criteria for the control participants were [1] being born preterm [2], having a first-degree relative diagnosed with a neurodevelopmental disorder [3], having a lifetime history of psychiatric, neurological, or learning disabilities.

This study was approved by the Swiss Ethics Committee for Human Research (CCER) and the Ethics Committee of the university of Geneva. Written consent was obtained from all participants. The parents of the 22q11DS participants, regardless of age, and the parents of the adolescents under 18 in the control group also had to sign the consent form.

### Material

All participants completed the oral language and autobiographical memory in one session.

### Oral Language narrative task

Linguistic narrative skills were tested using a classic ***storytelling task***, « Frog, where are you? » [26]. The story consists of 24 black and white images without text, spread over 15 pages. This story was chosen because it respects the typical structure of a story, consisting of an initial situation, a triggering event, twists and turns, a resolution, and an ending. Thus, it provides a very rich context for the production of language and mental states [27]. All the narratives were recorded for transcription. Macrostructure, microstructure, and pragmatic skills were analyzed.

A **macrostructure** grid analysis was adapted from previous studies in typically developing children, and in clinical settings such as children with specific language impairment or autism spectrum disorder [28, 29]. It aimed to explore the overall cohesion and informativeness of the narrative. A total of 61 items were included in the grid, collected from each event occurring during the different sequences of the story (introduction, triggering event, search of the frog, twists and turns, and resolution/conclusion), including semantic features and character presentations. One point was given for each item produced by the participant.

The **microstructure** analysis included several classical lexical and morpho-syntactic measures:

- The mean length of utterance (MLU), i.e. the mean number of words produced in each utterance.
- The number of different verb tense used in the narrative.
- The number of different words (content words) produced.
- The lexical diversity index (IDL), which corresponds to the ratio of the number of different words to the total number of words produced in the corpus.
- The corrected lexical diversity index (IDLC), calculated in the same way as the lexical diversity index, but considering only content words (and not prepositions, pronouns…).
- The number of morpho-syntactic errors: the pronoun, gender, preposition errors, the omissions, the inappropriate verb tenses, the ungrammatical sentences were recorded (1 point per error).

The **pragmatic analysis** grid was adapted from previous studies [30–32], and included the computation of emotion and inference:

- The number of mental verbs: These verbs can give an indication of the participant’s theory of mind: 1 point was awarded for each verbal reference to the mental and emotional content of the character.
- The reference to emotions: 1 point for each emotion evoked in the story (example: “the boy is happy”).
- The use of direct/indirect comment: 1 point awarded for each direct or indirect speech (example: “the boy said: frog, where are you?“).
- The number of hedges: 1 point awarded for each word indicating uncertainty (e.g. “maybe”, “probably”, etc.).
- The meta-comments: 1 point given for each comment indicating surprise or contradiction of the participant’s expectations (example: “but, too weird!“).
- The causal connectors: reflects the child’s ability to integrate information into the story to explain an emotion or behavior and allows the measurement of the pragmatic narrative cohesion of the discourse: 1 point awarded for each connector (example: “the boy is sad because the frog is gone”).

### Memory tasks

**Autobiographical episodic memory** was assessed using a paradigm including recollection of past personal memories and future thinking conditions which was suitable for 22q11DS participants [20] adapted from a previous task [33]. Participants were asked to recall past personal events (recollection condition) and to imagine plausible future events (future thinking or projection condition). For these *two temporal conditions* (past recollection and future projection), participants had to relate an event they had experienced or could experience, based on a cue word representing emotions (sad, scared, excited, relaxed). Participants were asked to provide as many details as possible about this event, including *sensory details* such as olfactory/gustatory, visual, and auditory details, as well as *non-sensory details* such as thoughts, feelings, and actions.

Participants were asked to tell 8 narratives: one narrative per condition (recollection and future thinking) for each emotional cue word (sad, scared, excited, relaxed). The order of words and conditions for each word was randomized. All responses were videotaped to carry out a double assessment of the narratives.

The type of autobiographical narrative (specific, extended, or categorical) was determined by whether participants were recounting an event [1] specific: events that took place in a specific place, on a specific day, and lasted no longer than one day; [2] extended: events that lasted longer than one day; [3] categorical: collection of events that were unrelated to each other or did not refer to a specific time period.

Measures included the percentage of past episodic memories and future projection narratives and the total number of sensory details or non-sensory details produced in the past/future conditions.

#### Standardized long-term verbal memory task

to test for potential difficulties in retrieving and recalling stories from memory, verbal memory was assessed using *standardized story recall memory tasks* (The Logical Memory I and II tasks of the Wechsler MEM Clinical Scale for participants over 16 years of age [34, 35], or the Immediate and Delayed Verbal Memory subtests of the Children’s Memory Scale for participants under 16 years of age [36, 37].

### Statistical analyses

IBM SPSS version 26 software was used for statistical analyses.

Tests for normality were performed on the experimental and neuropsychological data using the Shapiro-Wilk test. Group differences were analyzed using the non-parametric Mann-Whitney *U*-test for all data, because most of them were not distributed normally and because of the small sample of participants.

Spearman rank order correlations (rho’s) were used to examine the relationships between the memory and language measures. Benjamini-Hochberg correction for multiple comparisons was applied to all correlational analyses. Analyses with the whole group but also with each groups separated were performed.

Regression analyses were performed to determine the roles of linguistic skills but also the effects of age and verbal intellectual functioning (ICV) in autobiographical episodic memory.

## Results

### General neuropsychological data

Table 1 displays the general standardized neuropsychological assessment (compared to the control group).

**Table 1:** Results obtained in the standardized cognitive assessment: standard scores, indexes or Raw score (standard deviation) and statistical analyses for groups performance’s comparison

	22q11DS group (*n* = 15)	Control group (*n* = 15)	Significance Test	*p*-value
**Intellectual Efficiency** (WISC V/WAIS IV)				
FSIQ (mean(SD))	77.87 (9.8913)			
VCI (mean(SD))	88.33 (8.7885)			
PRI (mean(SD))	76.23 (10.7809)			
Matrix subtest (mean(SD))	SS = 6.4666 (2.7482)	SS = 11.00 (2.7775)	*t*(28)=-4.4936	0.000***
**Memory** (Story recall: MEM/CMS)				
Immediate recall (mean(SD))	SS = 9.66 (2.5542)	SS = 9.60 (2.6673)	*t*(28) = 0.0699	0.944
Delayed recall (mean(SD))	SS = 9.53 (2.5598)	SS = 8.86 (2.774)	*t*(28) = 0.6840	0.499

Within the Wechsler Intelligence Scale, the verbal comprehension index was situated in the low average range (mean VCI = 88.3, SD = 8.78). Perceptive reasoning index and global intellectual functioning were borderline (PRI = 76. 23; FSIQ = 77.8).

Regarding long-term verbal memory, the scores obtained in the immediate and delayed story recall of the standardized tasks (CMS/MEM) were situated in the average range for all participants and did not reveal statistically significant differences when comparing the clinical and control groups (*p* >.05).

### Linguistic narrative skills

**Fig. 1:**
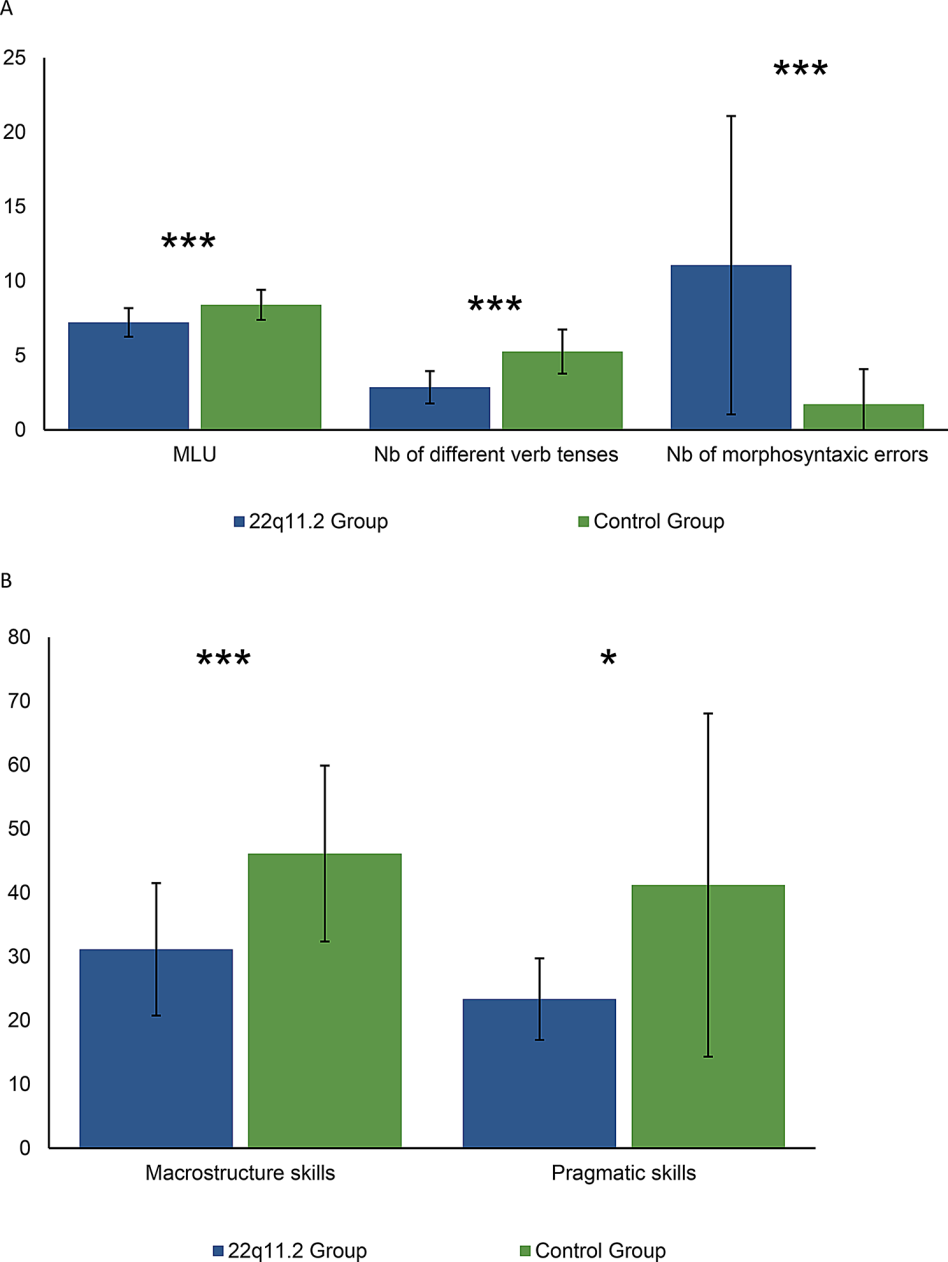
Microstructural (**A**), macrostructural and pragmatic (**B**) linguistic results obtained by the 22q11.2 and control groups (***= p <.01; *=p <.05)

Participants with 22q11DS displayed significantly lower scores in all linguistic levels, i.e. in the macrostructural (*p* <.001), pragmatic skills (*p* <.05) and in several microstructural measures (see below for detailed statistics) compared to the controls (Fig. 1).

Among the microstructural analyses, significant differences between both groups appeared for the mean length of utterance measure (*p* <.01), for the number of morphosyntactic errors produced (*p* <.0001) and the number of different verb tenses used (*p* <.0001). Lexical measures (IDL, IDLC, Number of different words produced) did not reveal statistically different results between the 22q11DS and control groups (Table 2).

**Table 2:** Results obtained in the linguistic (microstructure, macrostructure, pragmatics) and autobiographical memory (past recollection, future thinking) measures: mean scores (standard deviation) and statistical analyses for groups performance’s comparison

	22q11DS group (*n* = 15)	Control group (*n* = 15)	Mann Whitney U	Z score	*p*-value
**Microstructure linguistic skills**					
MLU (mean(SD))	7.22 (0.991)	8.40 (1.0473)	179.5	2.779	0.004**
Nb of morphosyntactic errors (mean(SD))	11.06 (10.0231)	1.73 (2.3513)	25.5	-3.65	< 0.001***
Nb of different verb tenses (mean(SD))	2.86 (1.1255)	5.26 (1.5337)	201.5	3.751	< 0.001***
Nb of different words (mean(SD))	143.13 (46.5286)	182.06 (53.4758)	160	1.97	0.050
IDL (mean(SD))	0.30 (0.0742)	0.31(0.045)	116.5	0.166	0.870
IDLC (mean(SD))	0.426 (0.0968)	0.432(0.0622)	121.5	0.374	0.713
**Macrostructure linguistic skills** (mean(SD))	31.13 (9.6427)	46.13 (6.3905)	201	3.674	< 0.001***
**Pragmatic linguistic skills** (mean(SD))	23.33 (13.3049)	41.2 (25.9594)	166.5	2.242	0.025*
**Autobiographical memory**					
% episodic narratives- past condition (mean(SD))	68.88 (29.7316)	91.07 (20.2818)	156.0	2.449	0.026*
% episodic narratives- future condition (mean(SD))	46.11 (34.4085)	83.93 (20.2818)	169.5	2.909	0.004**
Nb of sensory features - past condition (mean(SD))	1.7333 (2.1124)	5.4 (3.9123)	177.5	2.726	0.006**
Nb of sensory features – future condition (mean(SD))	1.0666 (1.1813)	4.9333 (5.0261)	173.5	2.58	0.01*
Nb of non-sensory features- past condition (mean(SD))	11.8 (4.9886)	14.2 (5.6592)	149	1.519	0.137
Nb of non-sensory features -future condition (mean(SD))	12.2 (5.0754)	10.6 (5.1871)	98.5	− 0.582	0.561

### Autobiographical episodic memory

The recollection of **autobiographical memories** (past condition) in the four conditions (sad, scared, excited, relaxed) was rated as episodic in nearly 70% of the narratives in the 22q11DS carrier group, which is significantly less (*p* <.05) than the percentage of episodic narratives obtained by the control group (higher than 90%). Moreover, the analyses showed a reduced number of sensory-perceptive details (but not non-sensory details) produced in the clinical group compared to the control group (*p* <.01) (Table 2).

***Episodic future thinking*** was difficult for the 22q11DS participants and only 46% of the narratives were rated as episodic events. This was significantly different from the percentage obtained by the controls (83%) (*p* <.05). Here again, the number of sensory details produced (but not non-sensory details) was significantly lower in the clinical group compared to the control group (*p* =.01) (Table 2).

### Correlations and regression analyses

#### Autobiographical memory: percentage of episodic narratives

When considering the whole group of participants, and after Benjamini-Hochberg correction for multiple comparisons, all linguistic levels, i.e. microstructure, macrostructure and pragmatic skills were significantly correlated (*p* <.05) with the percentage of episodic narratives produced in the future thinking condition. Macrostructure and pragmatic skills (*p* <.05) correlated with the percentage of episodic narratives produced in the past condition. However, no significant correlations were found between linguistic skills and autobiographical memory abilities when analyzing the results per group of participants (22q11 DS and control groups separated) and after controlling for multiple comparisons (Table 3). This suggests that the correlational obtained in the whole group were biased by the basic differences of linguistic and memory skills of each group.

**Table 3:** Spearman’s correlation analyses between the percentage of episodic autobiographical memories and linguistic narrative skills for the whole group and per separated group of participants [Benjamini-Hochberg adjusted *p*-values]

		Autobiographical memory (% episodic narratives)	
		Past recollection	Future thinking
Whole group of participants	**Linguistic skills**		
	Microstructure (Nf of morphosyntaxic errors) Macrostructure Pragmatics	*r*=-.302, *p* =.112 [*p* =.112] *r* =.403, *p* =.030* [*p* =.036]* *r* =.435, *p* =.018* [*p* =.027]*	*r*=-.439, *p* =.017* [*p* =.027]* *r* =.552, *p* =.002** [*p* =.012]* *r* =.483, *p* =.008** [*p* =.024]*
22q11 DS group	**Linguistic skills**		
	Microstructure (Nf of morphosyntaxic errors) Macrostructure Pragmatics	*r*=-.107, *p* =.705 [*p* =.943] *r*=-.186, *p* =.507 [*p* =.943] *r*=-.020, *p* =.943 [*p* =.943]	*r*=-.045, *p* =.875 [*p* =.943] *r* =.539, ***p*** =.04* [*p* =.132] *r* =.526, ***p*** =.044*[*p* =.132]
CTRL group	**Linguistic skills**		
	Microstructure (Nf of morphosyntaxic errors) Macrostructure Pragmatics	*r*=-.129, *p* =.660 [*p* =.747] *r* =.437, *p* =.118 [*p* =.354] *r* =.602, ***p*** =.023* [*p* =.138]	*r*=-.172, *p* =.557 [*p* =.747] *r* =.095, *p* =.747 [*p* =.747] *r* =.290, *p* =.315 [*p* =.63]

#### Autobiographical memory: number of sensory details

When considering the whole group of participants, the microstructure measure and the pragmatic score (*p* <.05) correlated with the number of autobiographical episodic sensory details produced in past recollection condition. However, no significant correlations were found between linguistic skills and the number of sensory details when analyzing the results per group of participants (22q11 DS and control groups separated) and controlling for multiple comparisons (Table 4). This suggests again that the correlational obtained in the whole group were biased by the basic differences of linguistic and memory skills of each group.

**Table 4:**
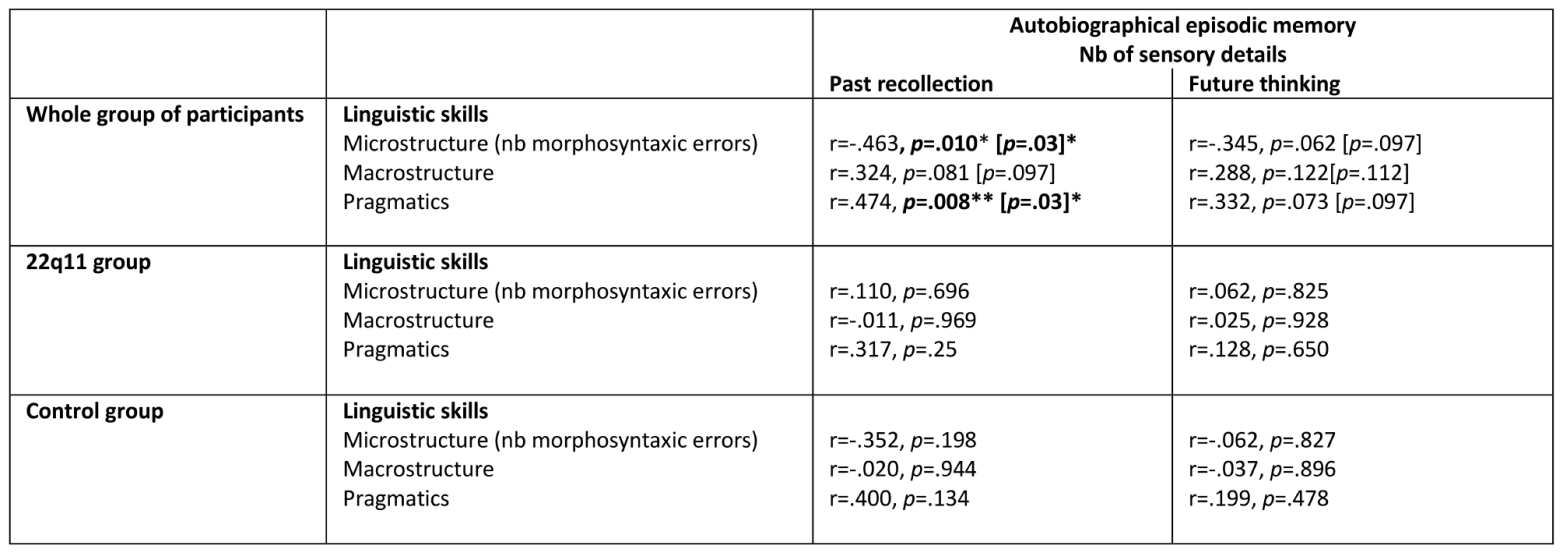
Spearman’s correlation analyses between the number of sensorial details reported in the autobiographical episodic memory and the linguistic skills for the whole group of participants and per separated group [Benjamini-Hochberg adjusted *p*-values]

#### Standardized verbal memory test

No correlations were found between the linguistic skills and the standardized verbal story-recall memory test (CMS/MEM).

#### Age and cognitive effects on autobiographical memory results in the 22q11DS group

Robust linear regression analyses showed a significant effect of age in the 22q11DS group on the percentage of episodic autobiographical narratives in the future condition (*t* [13] = 2.322, *p* =.039) but not in the sensory/non-sensory findings. An effect of verbal intelligence functioning (VCI) was observed only in the future projection condition and on the number of sensory details (*t* [13] = 4.137, *p* =.001) but not for the number of non-sensory details (*t* [13] = 0.015, *p* =.988).

## Discussion

Overall, the results revealed poor narrative abilities in young adults with 22q11SD, affecting all linguistic levels, i.e. microstructure, macrostructure, and pragmatic components of the “frog story” telling task. Regarding autobiographical memory, both recollection of memories and future projection narratives lacked episodic features, and finer-grained analyses revealed that sensory/perceptual details were mostly missing in 22q11DS (but not non-sensory details) compared to controls. When considering the specific involvement of language skills in autobiographic episodic memory, it appeared that the level of linguistic mastery did not account for the autobiographical memory skills, either in the 22q11DS or in the control groups.

To the best of our knowledge, this is the first study to report on the **linguistic narrative abilities** in adolescents and young adults with 22q11DS, including detailed analyses of the macrostructure, microstructure, and the pragmatic components of the narration in a picture storytelling task. Contrary to previous studies reporting normalized lexical, morpho-syntactic and narrative skills in late childhood [16, 18], this study shows that verbal production remains clearly suboptimal in young adults with 22q11DS when higher-order, more complex linguistic skills such as narration are required, even in individuals with a normal verbal intellectual quotient. Indeed, poorer mastery of all language levels (macrostructure, microstructure, and pragmatics) was observed in 22q11DS carriers compared to controls. Global coherence between the different phases of the story was poor (macrostructural impairment), inferences and reference to the feelings, emotions and thoughts of the characters were less frequent, suggesting lack of theory of mind (pragmatic impairment) in the clinical group. At the microstructural level, 22q11DS carriers struggled mainly with complex morphosyntactic skills, as their verbal productions contained more syntactic errors, were characterized by shorter sentences (as measured by mean length of utterance) and contained less variety in verb tense compared to the control group. Lexical measures, however, seemed to be preserved. These results provide strong evidence for a long-lasting specific language deficit in the 22q11.2 deletion syndrome.

Regarding **long-term memory skills**, the 22q11DS group obtained average scores on the standardized declarative verbal memory assessment, i.e. the story recall task, and their results did not differ from the control group, as has been shown in previous studies using rote verbal memory recall tasks [22, 23, 38]. This rules out the hypothesis of global story recall deficits in 22q11.2 carriers. Furthermore, it appears that the poor narrative language skills in young adults with 22q11DS do not affect their immediate and delayed story recall, in contrast to children with developmental language disorders who typically present with impaired verbal memory [39, 40]. Note however that declarative verbal memory difficulties in children with DLD appeared to be significantly modulated by their working memory skills and/or nonverbal IQ. Here, no correlations were found between narrative skills and declarative standardized verbal memory.

Whereas immediate and delayed story recall were preserved in the 22q11DS participants, ***autobiographical memory*** narratives often consisted of summarizing the central elements of a particular event, with minimal detail, or describing the general framework extracted from similar experiences. This failure to provide episodic features in autobiographical memories affected both the past recollection and the future projection conditions. In addition, further analyses revealed that the 22q11DS participants reported very few sensory (but not non-sensory) details compared to the control group, suggesting specific difficulties in accessing perceptual features (rather than thoughts/feelings) of the recalled or imagined scenes. These autobiographical narratives could be considered as « gist » memory or schema narratives rather than true episodic memories, and may reflect an impaired cerebral network connecting the posterior hippocampus, where rich and detailed personal memories are recalled, to the posterior neocortex, where the perceptual representations are stored [41, 42]. Interestingly, reductions in posterior temporo-parieto-occipital brain volumes have been found in 22q11DS carriers (see meta-analysis: [43, 44]. Furthermore, specific alterations of the hippocampi have also been demonstrated in this syndrome [43, 45–48] providing congruent arguments in favor of the hypothesis of a dysfunctional network linking the hippocampi to the posterior cerebral cortex, accounting for the episodic autobiographical memory disorders and specific impairment in accessing perceptual features, as highlighted in this study.

Interestingly, while both the past recollection and future episodic projection conditions were impaired in 22q11DS participants compared to the control group, the poor narrative skills observed in the clinical group did not account for the lack of episodic features in autobiographical memories (measured by the percentage of episodic narrative produced in both temporal conditions). Moreover, age and intellectual functioning did not account for the poor autobiographical recollection of their (past) memories either. Thus, 22q11.2 carriers may indeed have specific autobiographical memory deficits.

Linguistic skills did not account either for the future episodic projection skills, but age and intellectual functioning contributed to the number of details or to the percentage of episodic future thinking. Thus, future thinking may rely on more complex variables and may be more sensitive to developmental factors such as mental age and cognitive maturity as has been shown in other studies [49]. It remains unclear whether future thinking relies on scene construction skills and/or on self-projection skills. Indeed, future thinking requires scene construction skills and both the scene construction skills and the ability to imagine the scenes seem to rely on medial temporal lobe processing [50]. However, future thinking also requires increased self-awareness, or self-referential processing. Self-referential processing, or self-concept develops during adolescence and appears to be supported by the ventromedial prefrontal cortex and medial posterior parietal cortex in typically developing teenagers [51]. In a functional magnetic resonance imaging study (fMRI), 22q11DS carries were found to have hypo-activations in several cerebral areas compared to controls during a self-referential processing task, suggesting atypical brain recruitment during self-concept formation in this population [52]. Finally, future episodic autobiographical thinking requires imagination and creative thinking, which may be an additional barrier, as 22q11DS carriers often have autistic features. Potentially poor self-referential processing and/or poor scene construction skills, together with reduced creative thinking/imagination, may thus contribute to poor episodic autobiographical future thinking in 22q11DS carriers. This would be consistent with the finding of several studies reporting and arguing for the additive contribution of multiple cognitive skills to episodic autobiographical memory in children aged 1 ½ to 16 years [53].

The 22q11DS participants included in this study benefitted from relatively good intellectual functioning compared to what is usually reported in this syndrome. An important and interesting point of this study is precisely to show that despite (low-) average verbal intellectual functioning (mean VCI ~ 88), young adults with 22q11.2DS anyway struggle with linguistic, narrative skills. Another point of interest is that contrary to our hypotheses, this poor linguistic mastery does not account for the poor episodic autobiographical memories in this clinical group. As our sample was rather small and the age range relatively large, these findings should be extended and replicated in larger 22q11DS populations in further studies. Moreover, these findings require a further exploration of the functional and social impact of these narrative and autobiographical memory impairments.

## Conclusion

First, this study demonstrated that adolescents and young adults with 22q11DS still struggle with high-level language skills such as storytelling tasks, with impairments affecting all linguistic levels, i.e., the microstructural, macrostructural, and pragmatic components of narrative. Second, impaired episodic autobiographical recollection of personal memories was observed in 22q11DS carriers compared to controls, together with reduced access to sensory (and not non-sensory) details.

These autobiographical episodic memory deficits seemed to be independent of the linguistic weakness and could reflect true dysfunctional memory networks between the hippocampi and the posterior cortical areas, where alterations have indeed been reported in this syndrome.

## Data Availability

No datasets were generated or analysed during the current study.
